# Auricular Acupuncture and Cognitive Behavioural Therapy for Insomnia: A Randomised Controlled Study

**DOI:** 10.1155/2016/7057282

**Published:** 2016-05-08

**Authors:** L. Bergdahl, J.-E. Broman, A. H. Berman, K. Haglund, L. von Knorring, A. Markström

**Affiliations:** ^1^Psychiatry, Department of Neuroscience, Uppsala University, 751 85 Uppsala, Sweden; ^2^Lung, Allergy and Sleep Research, Department of Medical Sciences, Uppsala University, 751 85 Uppsala, Sweden; ^3^Department of Clinical Neuroscience, Center for Psychiatry Research, Karolinska Institutet, Norra Stationsgatan 69, 113 64 Stockholm, Sweden

## Abstract

*Objective*. The most effective nonpharmacological treatment for insomnia disorder is cognitive behavioural therapy-insomnia (CBT-i). However CBT-i may not suit everyone. Auricular acupuncture (AA) is a complementary treatment. Studies show that it may alleviate insomnia symptoms. The aim of this randomised controlled study was to compare treatment effects of AA with CBT-i and evaluate symptoms of insomnia severity, anxiety, and depression.* Method*. Fifty-nine participants, mean age 60.5 years (SD 9.4), with insomnia disorder were randomised to group treatment with AA or CBT-i. Self-report questionnaires, the Insomnia Severity Index (ISI), Dysfunctional Beliefs and Attitudes about Sleep scale (DBAS-16), Epworth Sleepiness Scale (ESS), and Hospital Anxiety and Depression scale (HAD), were collected at baseline, after treatment, and at 6-month follow-up. A series of linear mixed models were performed to examine treatment effect over time between and within the groups.* Results*. Significant between-group improvements were seen in favour of CBT-i in ISI after treatment and at the 6-month follow-up and in DBAS-16 after treatment. Both groups showed significant within-group postintervention improvements in ISI, and these changes were maintained six months later. The CBT-i group also showed a significant reduction in DBAS-16 after treatment and six months later.* Conclusions*. Compared to CBT-i, AA, as offered in this study, cannot be considered an effective stand-alone treatment for insomnia disorder. The trial is registered with ClinicalTrials.gov NCT01765959.

## 1. Introduction

Insomnia disorder is a common and growing problem, which is approximately twice as common for women as for men [1]. Aside from unsatisfactory sleep quality, insomnia disorder is diagnostically characterised by difficulty in falling asleep, involuntary and early awakenings more than three times a week for more than three months, and daytime impairment affecting important cognitive functions [2]. Insomnia disorder causes personal suffering and increased societal costs. On an individual level it leads to increased risk of other comorbidities such as anxiety and depression [3]. In addition, insomnia disorder increases the risk of developing cardiovascular disease [4], type 2 diabetes, obesity [5, 6], and a weakened immune system [7]. It may also increase the risk of substance dependence [8]. On a societal level the largest insomnia-related costs are represented by increased sick leave, loss of production [9, 10], and traffic and fall accidents [8, 11].

Treatments for insomnia disorder can be pharmacological, nonpharmacological, or a combination of both. Nonbenzodiazepines, also known as z-drugs, are a common pharmacological treatment of insomnia disorder [8]. Although pharmacological treatment may be effective, there may also be side effects, such as drug dependence and residual daytime sedation. Short-term usage is the state-of-the-art treatment for z-drugs. However despite these recommendations there are patients receiving the treatment for a long time.

In the present study, the target population was persons who had used z-drugs for more than six months and experienced an unsatisfactory treatment outcome. Insomnia research over the last two decades shows a trend towards increased nonpharmacological interventions, where behavioural therapies are emphasised [12]. The most effective nonpharmacological treatment of insomnia disorder is cognitive behavioural therapy (CBT) [8, 13]. CBT is a psychotherapeutic treatment method which helps the patient actively work to change thoughts, emotions, and behavioural patterns that do not promote health. CBT for insomnia (CBT-i) consists of sleep-focused manual-based treatment sessions encompassing stimulus control, sleep restriction, cognitive strategies, relaxation techniques, and sleep hygiene (e.g., [14]). The treatment goal is to identify and change the behavioural, psychological, and physiological factors that maintain the insomnia. However CBT-i may not be a suitable treatment method for everyone. According to a review by Harvey and Tang [15] CBT seemed less effective for treating insomnia disorder compared to treating other psychological disorders. To refine CBT-i they call for further experimental investigations, further theory development, and clinical practice. Montserrat Sánchez-Ortuño and Edinger [16] hypothesise that there are subgroups among patients with insomnia disorder that may respond differently to manual-based CBT. For instance, worried and symptom-focused patients seemed to benefit more from CBT than patients who were worried and medication biased.

Acupuncture is a nonpharmacological treatment method based on complementary medicine, for which interest has increased over the last years. It has, to some extent, been introduced in the ordinary health care system [17]. Acupuncture has been used to treat insomnia disorder [18, 19] and according to a review by Huang et al. [20] acupuncture may influence the autonomic nervous system [20]. Positive findings have also been reported for acupuncture in treatment of anxiety [21] and depression [22]. Auricular acupuncture (AA) is a branch of acupuncture where needles are inserted in different areas in the outer ears [23]. An earlier, randomised trial found modest improvements over time on a variety of sleep parameters for women in psychiatric care randomised to AA according to standard or sham protocols, with fewer awakenings in the standard treatment group [24]. Prisco et al. [25] performed a randomised trial in a military population with posttraumatic stress disorder (PTSD) and sleep disturbances that suggested that AA has a beneficial short-term effect on sleep quality compared to sham control group and waiting-list [25]. A meta-analysis by Cheuk et al. [18] including 33 studies, where different forms of acupuncture were used to treat insomnia disorder, indicated that AA may ameliorate sleep quality compared to placebo. However due to the variation in methodological quality of the studies included, such as small samples or lack of randomisation and/or control groups, no certain conclusions could be drawn. Further research with more stringent methodology was recommended to assess AA effectiveness for insomnia disorder [18].

**Figure 1:**
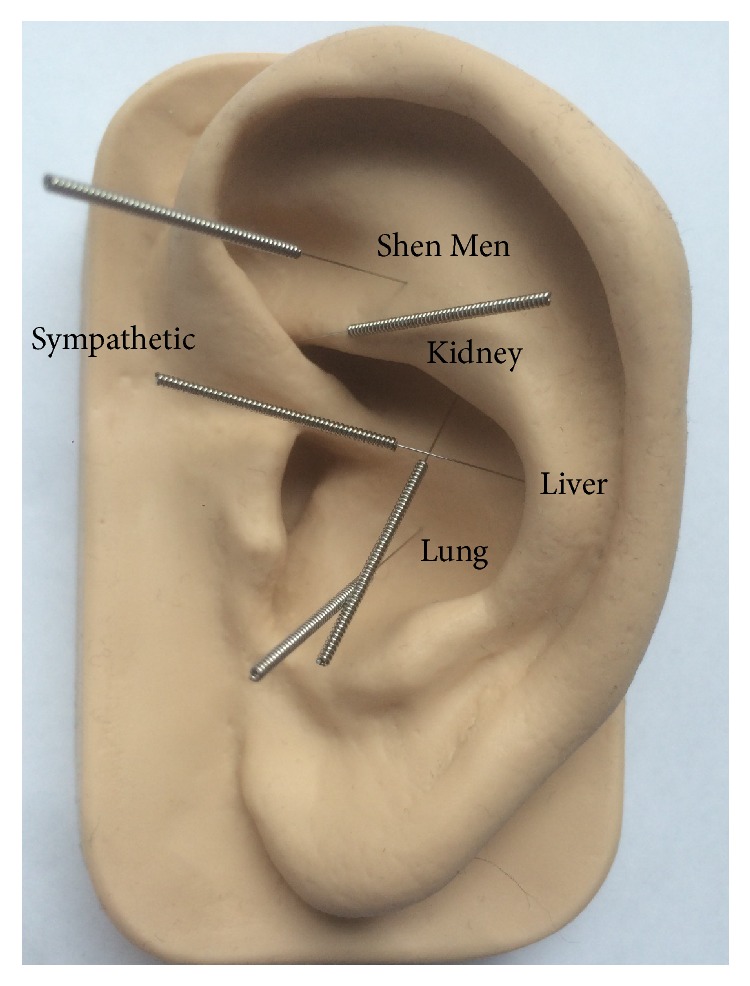
Acupuncture points according to the NADA protocol: Shen Men, sympathetic, kidney, liver, and lung.

AA has also been used within psychiatric care for substance dependence (e.g., [26, 27]), where a standardised insertion pattern, defined as the NADA (National Acupuncture Detoxification Association) protocol, is normally practised. The NADA protocol is a five-point setting, using the European ear map of Paul Nogier, and was developed during the late 1970s and mid-1980s. The point selection is sympathetic, Shen Men, liver, kidney, and lung [28, 29] (see Figure 1). Bergdahl et al. performed a qualitative study at an outpatient clinic for substance dependence where AA according to the NADA protocol was administered to patients suffering from post-acute withdrawal symptoms. Apart from the participants' subjective sense of increased relaxation and well-being, the results from the interviews showed that many of the participants also gained a subjective higher sleep quality [26]. This raised the question as to whether the results would be transferable to treatment of insomnia disorder. Hence, the purpose of the present study was to add further clarity regarding AA as a treatment for insomnia disorder.

*Aim*. The primary aim of this study was to compare treatment effects of AA with CBT-i and evaluate symptoms of insomnia severity for short- and long-term perspectives. Symptoms of anxiety and depression were also evaluated.

## 2. Method

This was a prospective randomised controlled study (RCT) evaluating immediate and long-term treatment effects.

### 2.1. Participants

Inclusion criteria covered men and women (18–75 years) with insomnia disorder, diagnosed according to the DSM-5 [2], who had been using nonbenzodiazepine hypnotics at least three times a week for six months or more but still had maintained insomnia symptoms. They also had a wish to end their usage of sleep medication. Comprehension of the Swedish language was required. Exclusion criteria were substance dependence (alcohol or drugs), patients with severe psychiatric disorder, severe somatic disease, pharmacological treatment with antipsychotic drugs and/or morphine/morphine-like drugs, patients who had initiated antidepressant or anxiolytic treatment within the past 3 months, or pregnancy.

The final sample comprised 50 women and 9 men (*N* = 59) who had had insomnia for more than six months and despite pharmacological treatment had residual insomnia symptoms and wanted to discontinue their medication. They were included between January 2013 and October 2014. The participants' mean age was 60.5 (SD 9.4). Twenty-six participants were retired, twenty-nine were working, and one was studying. Three participants were on long-term sick leave and/or had disability pension and one participant was unemployed.

### 2.2. Procedure

**Figure 2:**
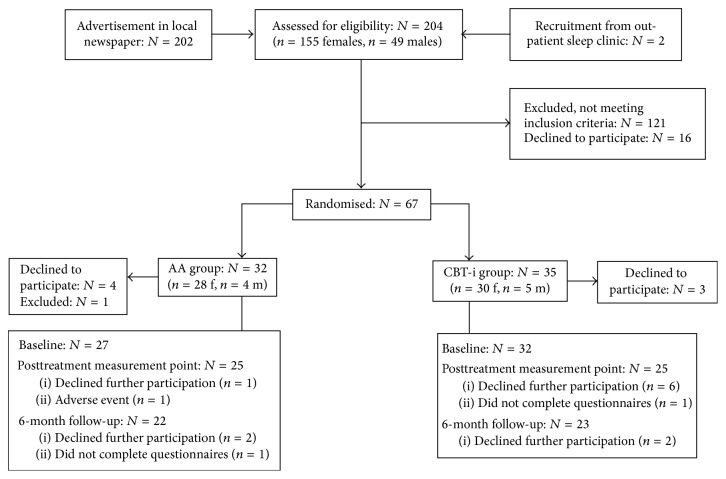
Participant flow diagram and passage of events during the study.

Participants were recruited by advertisement in the local newspaper and from an outpatient sleep clinic. Regarding the recruitment by the advertisement, the subjects initiated the first contact. At this point, during which the first screening of inclusion/exclusion criteria also took part, the subjects were given oral and written information about the study by the first author (LB). The subjects from the outpatient sleep clinic were recruited by the senior author (AM). All were informed that participation was voluntary and that they could withdraw from the study at any time without any negative consequences. Inclusion was made at a clinical visit with AM, an experienced medical sleep specialist, who assessed all subjects before inclusion. At this point the consent form was signed after which the participants were included in the study. After inclusion the participants were randomised to group treatment with AA or CBT-i (see participant flow chart in Figure 2). The randomisation procedure was carried out by means of a prestudy randomisation list. Block randomisation was used where each group contained five positions for each treatment. The investigators were informed of each individual randomisation and the participants were coded according to the groups and registered in the medical chart. Measurements were made at baseline, after intervention, and at 6-month follow-ups, using self-report questionnaires.

### 2.3. Interventions

#### 2.3.1. AA

The AA group received treatment according to the NADA protocol twice a week for four weeks. All treatments were carried out in hospital facilities and were performed by two AA- trained members of the psychiatric medical staff. Both members had undergone acupuncture training according to the NADA protocol.

During each session, the participants were treated with five acupuncture needles in each of the outer ears for 45 minutes; no needle stimulation was performed. During the treatment the participants sat in chairs and were instructed by the acupuncturist to close their eyes and to focus on keeping their breathing calm and regular. The acupuncturists aimed to have the same attitude and behaviour in order to make the treatment as similar as possible for all participants. When the needles had been inserted the acupuncturist left the room.

#### 2.3.2. Equipment

Disposable, sterile stainless Zhongyan Taihe acupuncture needles (0.18 × 13 mm) were used. Before the needles were inserted the acupuncturists' hands and the participants' outer ears were disinfected with disinfectant solution.

#### 2.3.3. CBT-i

The CBT-i group received manual-based group treatment [30], focused on cognitive restructuring, once a week for six weeks. The sessions contained information regarding sleep physiology, different ways of coping with sleeping problems, sleep restriction, maintaining factors, stimulus control, and relaxation techniques. Each session lasted for 90 minutes. Three registered psychologists who all had undergone CBT training and were experienced in giving CBT-i treatment carried out the treatments. All sessions were performed in hospital facilities.

### 2.4. Questionnaires

#### 2.4.1. The Insomnia Severity Index (ISI)

ISI is a seven-item questionnaire assessing the severity of insomnia as regards sleep onset and maintenance of sleep, satisfaction with current sleep patterns, interference with daily functioning, impairment due to sleep problems, and level of distress caused by insomnia disorder. The rating scale is 0–4 for each item with a total score range from 0 to 28. A higher score indicates higher severity of insomnia symptoms [31]. A change score in ISI of seven is suggested as a moderate improvement of insomnia symptoms whereas a change score of nine is regarded as marked improvement [32]. ISI was used as the primary outcome measure.

#### 2.4.2. The Dysfunctional Beliefs and Attitudes about Sleep Scale (DBAS-16)

Previous studies confirm an association between the level of insomnia severity and dysfunctional beliefs about sleeplessness [33]. DBAS-16, a shorter version of DBAS-30, is a 16-item questionnaire with response alternatives from 0 to 10. The sum of the items is divided by 16 and the cut-off score is 3.8. A high result indicates more severe dysfunctional thoughts and beliefs [34]. In this study, DBAS-16 was used to detect subjective beliefs, worries, and expectations concerning sleep, sleeplessness, and sleep medication [35] among the participants.

#### 2.4.3. The Epworth Sleepiness Scale (ESS)

In order to measure daytime sleepiness ESS was used. Eight questions, graded from 0 to 3, describe everyday situations that may induce sleepiness. The score range is 0–24 where a score of more than 10 is regarded as excessive daytime sleepiness [36].

#### 2.4.4. The Hospital Anxiety and Depression Scale (HAD)

Finally, HAD was used to detect and evaluate symptoms of anxiety (HAD-A) and/or depression (HAD-D). These scales have seven items with a rating from 0 to 3 and a total score of 21 where a score above 8 indicates the presence of anxiety or depression [37].

### 2.5. Statistical Analysis

The skewness ratio (skewness value divided by the standard error) was used to check for normality in the outcome instruments for each treatment group at all three session points. Only ISI and ESS at baseline for the AA group showed a skewness ratio exceeding ±2 indicating that the majority of measures showed satisfying normality. To examine treatment effects over time a series of linear mixed models, using the Restricted Maximum Likelihood method and unstructured variance components, were estimated with the ISI, ESS, DBAS-16, and HAD questionnaires as outcome variables. Treatment group, gender, session point, time on medication, age, and the interaction of treatment group and session point were chosen as fixed factors/covariates. Dropouts were assessed using Pearson Chi-Square test and Mann-Whitney test. A significance level of 5% was used. All analyses were performed with SPSS 21.

### 2.6. Baseline Data

The groups did not differ significantly from each other on ISI at baseline measurements; nor were there any differences between the participants and the dropouts. In total 59 participants were analysed at baseline, 27 in the AA group and 32 in the CBT-i group.

### 2.7. Ethics

The project was approved by the Regional Ethical Vetting Board in Uppsala (ref number 2012/353) and was registered in the ClinicalTrials.gov database (ClinicalTrials.gov ID: NCT01765959).

## 3. Results

**Figure 3:**
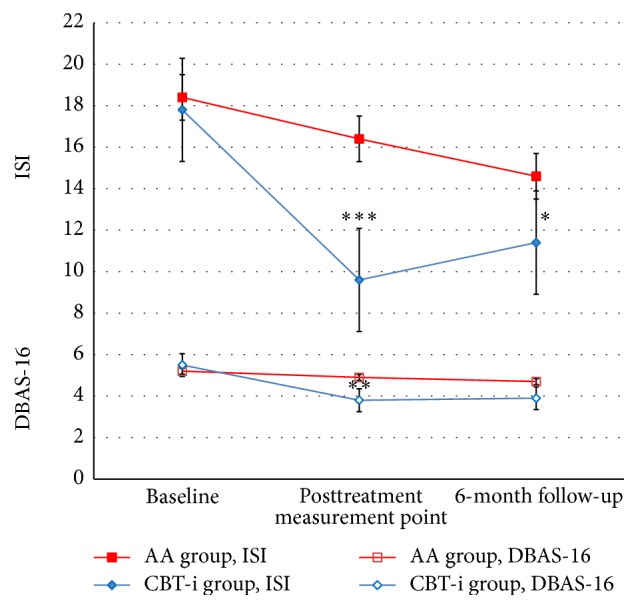
Changes in ISI and DBAS-16 scores between groups from baseline to posttreatment measurement point and from baseline to 6-month follow-up. ^*∗*^ *p* < 0.05, ^*∗∗*^ *p* < 0.01, and ^*∗∗∗*^ *p* < 0.001. ISI: Insomnia Severity Index; DBAS-16: Dysfunctional Beliefs and Attitudes about Sleep scale.

The AA group was compared to the CBT-i group and the results were analysed to reveal changes over time from baseline to posttreatment measurement point and from baseline to the 6-month follow-up. Significant between-group improvements occurred in favour of CBT-i in the primary outcome measure, ISI, at the posttreatment measurement point (*p* < 0.001), and at the 6-month follow-up (*p* < 0.05). In DBAS-16 there was a significant change in favour of the CBT-i group at the posttreatment measurement point (*p* < 0.01); however the result was not maintained at the 6-month follow-up. The between-group parameter estimate (PE) after treatment and at the 6-month follow-up was 6.28 (*p* < 0.001) and 2.95 (*p* < 0.05) for the ISI and 1.51 (*p* < 0.01) and 1.29 (*p* < 0.05), respectively, for DBAS-16. For HAD-D there were no differences between the groups since both groups showed significant improvements. None of the groups showed improvements in HAD-A or ESS. Comparisons between the groups are presented in Figure 3.

Within-group results are presented in Table 1.

### 3.1. CBT-i

ISI and DBAS-16 scores declined from baseline to posttreatment measurement point (*p* < 0.001,  *p* < 0.001) and from baseline to the 6-month follow-up (*p* < 0.001,  *p* < 0.001). The PE for ISI were −8.16 (*p* < 0.001) after treatment and −6.09 (*p* < 0.001) at the 6-month follow-up. For DBAS-16 the PE were −1.80 (*p* < 0.001) after treatment and −1.76 (*p* < 0.001) at the 6-month follow-up.

Clinically significant posttreatment improvements measured by ISI showed that the CBT-i group had three (12%) cases with moderate improvement and thirteen (52%) cases with marked improvement. At the 6-month follow-up there were two (9%) cases with moderate improvement and nine (39%) cases with marked improvement. HAD-D scores also declined significantly from baseline to the 6-month follow-up (*p* < 0.05), PE −0.99 (*p* < 0.05). No improvements were seen in HAD-A or ESS.

### 3.2. AA

ISI scores declined at the posttreatment measurement point (*p* < 0.05), PE −2.07 (*p* < 0.05), and at the 6-month follow-up (*p* < 0.001), PE −3.27 (*p* < 0.001). Clinically significant posttreatment improvements measured by ISI showed zero cases of moderate improvement and two (8%) cases of marked improvement. At the 6-month follow-up there was one (5%) case with moderate improvement and there were three (14%) cases with marked improvement. HAD-D scores declined significantly from baseline to the 6-month follow-up (*p* < 0.05), PE −0.70 (*p* < 0.05). No significant changes were seen in DBAS-16, HAD-A, or ESS.

## 4. Discussion

The purpose of this study was to compare the treatment effects of AA with CBT-i to evaluate whether AA is a suitable treatment for insomnia disorder. The main finding is that CBT-i was superior to AA in decreasing insomnia symptoms and to some extent also in changing dysfunctional beliefs about sleep after the treatment and at the 6-month follow-up.

The primary outcome measure, ISI, showed clinically significant improvements according to the cut-off scores of moderate and marked improvements (i.e., change scores of seven and nine, resp.) as suggested by Morin et al. [32]. In the CBT-i group, 12% showed moderate improvement and 52% showed a marked improvement at the posttreatment measurement point. At the 6-month follow-up 9% maintained the moderate improvement and 39% maintained the marked improvement. At the posttreatment measurement point in the AA group there were no moderate improvements; however, 8% showed a marked improvement. At the 6-month follow-up 5% showed moderate improvement and 14% were markedly improved. Notably, in comparison to the posttreatment measurement point, some of the AA participants had improved their insomnia severity six months after the intervention. We speculate that the decrease in ISI in the AA group may have been due to an increased sense of relaxation or a decreased stress level, which may have led to subjectively improved sleep. It is however unclear if the changes in the AA group were due to an actual treatment effect or occurred by chance. These changes might also have occurred in a group receiving sham acupuncture or in a waiting-list group; the placebo effect of being included in a study may have been a confounding factor here. A wait-listed or Treatment As Usual (TAU) control group in this study might have elucidated whether or not the results in the AA group were due to the actual treatment or if the effect was random. Thus, future studies could have a third arm with a wait-list or placebo group.

Dysfunctional beliefs concerning sleep, sleep medication, and consequences of insomnia are a common feature for persons suffering from insomnia disorder and traditionally the DBAS-16 is an effective measure for CBT-i outcomes. CBT requires questioning one's behaviour in order to achieve behavioural changes. In accordance with previous findings [35, 38, 39], our results confirm CBT-i effectiveness for decreasing insomnia severity and dysfunctional beliefs about sleep. In this study, DBAS-16 was also used to measure potential changes in the AA group. However no changes were seen; AA participants were not provided with additional coping strategy training for cognitive restructuring and no change was seen in comparison to the CBT-i group.

As mentioned previously, insomnia disorder may give rise to anxiety and/or depression. These disorders may also give rise to insomnia disorder. In this study HAD was not used to diagnose anxiety and/or depression but to detect the occurrence of symptoms thereof. Both groups showed a statistically significant reduction of depressive symptoms measured by HAD-D six months after the intervention, but as a group the participants were not classified as clinically depressed.

## 5. Limitations

Small sample sizes are always an issue and are indeed a limitation. This should be kept in mind when interpreting the results. In addition, this study contains a low number of male participants, which is a limitation in the sense that there is no way to detect potential gender differences in the sample. Another limitation may be that no measurements of the treatment preferences and expectations were performed. This would have been of value since there were participants in both groups who were dissatisfied with the group allocation, which may have affected the credibility of the treatment results. One further limitation was that the CBT-i and the AA were administered in different dosages as regards time spent in therapy.

## 6. Conclusion

In this study auricular acupuncture was compared to CBT-i, which is regarded as the most effective nonpharmacological treatment for insomnia disorder. Compared to CBT-i, auricular acupuncture, as offered in this study, cannot be considered an effective stand-alone treatment for persons with insomnia disorder.

## Figures and Tables

**Table 1:** Linear mixed models with within-group effects for AA and CBT-i. Outcome variables are Insomnia Severity Index (ISI), Dysfunctional Beliefs and Attitudes about Sleep scale (DBAS-16), Hospital Anxiety and Depression scale (HAD-A, HAD-D), and Epworth Sleepiness Scale (ESS).

	Baseline	Posttreatment measurement point	6-month follow-up	Baseline versus posttreatment measurement point	Baseline versus 6-month follow-up
	Estimate (SE)	Estimate (SE)	Estimate (SE)	Estimate (SE)	Estimate (SE)
*ISI*					
AA	18.56 (0.70)	16.49 (0.94)	15.19 (0.94)	−**2.07 **(0.78)^**∗**^	−**3.27 **(0.84)^**∗∗**^
CBT-i	17.75 (0.65)	9.6 (1.04)	11.66 (1.25)	**−8.16 **(1.18)^**∗∗**^	−**6.09 **(1.33)^**∗∗**^
*DBAS-16*					
AA	5.20 (0.29)	4.83 (0.27)	4.70 (0.33)	−37 (0.29)	−50 (0.31)
CBT-i	5.55 (0.25)	3.74 (0.34)	3.79 (0.44)	−**1.80 **(0.34)^**∗∗**^	−**1.76 **(0.39)^**∗∗**^
*HAD-A*					
AA	5.58 (0.46)	4.69 (0.59)	5.22 (0.66)	−0.90 (0.46)	−0.36 (0.54)
CBT-i	5.93 (0.58)	5.24 (0.55)	5.32 (0.58)	−0.68 (0.54)	−0.61 (0.56)
*HAD-D*					
AA	5.33 (0.56)	4.94 (0.46)	4.63 (0.50)	−0.39 (0.40)	−**0**.**70 **(0.37)^**∗**^
CBT-i	5.78 (0.49)	5.29 (0.56)	4.78 (0.51)	−0.49 (0.63)	−**0**.**99 **(0.49)^**∗**^
*ESS*					
AA	4.56 (0.72)	3.75 (0.61)	4.44 (0.49)	−0.80 (0.54)	−0.12 (0.56)
CBT-i	5.12 (0.67)	4.85 (0.72)	4.65 (0.68)	−0.28 (0.71)	0.48 (0.57)

^*∗*^ *p *< 0.05, ^*∗∗*^ *p *< 0.001.
